# Determinants of SARS-CoV-2 Impacts on Small-Scale Commercial Broiler Production Systems in Egypt: Implications for Mitigation Strategies

**DOI:** 10.3390/ani11051354

**Published:** 2021-05-10

**Authors:** Assem Abu Hatab, Zhen Liu, Asmaa Nasser, Abourehab Esmat

**Affiliations:** 1 Department of Economics, Swedish University of Agricultural Sciences, 750 07 Uppsala, Sweden; 2 Department of Economics & Rural Development, Arish University, Al-Arish 45516, Egypt; 3 School of Business, Nanjing Normal University, Nanjing 210023, China; 4 Independent Consultant, Helwan 11731, Egypt; asmaaa.an@gmail.com; 5 Department of Agricultural Economics, Al-Azhar University, Assiut 71515, Egypt; abourehabesmat@azhar.edu.eg

**Keywords:** COVID-19, small-scale broiler production, poultry sector, polychoric principal component analysis, ordered logit model, Egypt

## Abstract

**Simple Summary:**

The COVID-19 pandemic has exerted a substantial impact on small-scale broiler production systems in developing countries and put their supply chains at risk of disruption. Drawing on a survey of 205 small-scale commercial broiler farms (SCBFs) in Egypt, this study identifies the primary pathways through which the pandemic has affected these farms and investigates the determinants of their perception of COVID-19 effects. The empirical results revealed that the pandemic affected SCBFs heterogeneously based on their management and production systems and resource endowment. In particular, individually owned farms and those with membership of poultry producer organizations and larger total asset values perceived significantly fewer effects. In addition, SCBFs operating in both local and provincial markets were less likely to perceive negative effects from the pandemic. Despite that the adoption of strict containment measures was essential for protecting public health, our results indicate that policy responses to COVID-19 must consider the likely effects on small businesses such as SCBFs since disruptions to such socioeconomically important supply chains will intensify human suffering from the pandemic. These findings of our study provide important implications for enhancing the preparedness and resilience of small-scale broiler production systems in developing countries to future pandemics and natural hazards.

**Abstract:**

As in many other countries, the outbreak of the COVID-19 pandemic, together with subsequent government containment measures, posed significant challenges to small-scale broiler production systems in Egypt. Based on a survey of 205 specialist small-scale commercial broiler farms (SCBFs) consisting of both farm-based and household-based production systems, this study identifies the primary pathways through which COVID-19 has affected SCBFs and investigates the determinants of farm perception of these effects. A polychoric principal component analysis sorted the effects of the pandemic on the SCBFs surveyed into five categories, namely, input availability, production and operational costs, labor and human resources, consumer demand and sales, and farm finances. Next, five ordered logit models were constructed to examine the determinants of the SCBFs’ perception of each category of these effects. Generally, the empirical results revealed that COVID-19 affected SCBFs heterogeneously based on their management and production systems and resource endowment. Female-led and household-based SCBFs perceived significantly greater COVID-19 effects. In contrast, individually owned farms and those with membership of poultry producer organizations and larger total asset values perceived fewer effects. In addition, SCBFs operating in both local and provincial markets were less likely to perceive negative effects from the pandemic on their broiler farming activities. Although the adoption of strict and immediate containment measures was essential for controlling the virus and protecting public health, our results indicate that policy responses to COVID-19 must consider the likely effects on small businesses such as SCBFs since disruptions to such socioeconomically important supply chains will intensify human suffering from the pandemic. Overall, our findings provide important implications for the formulation of effective strategies for mitigating the impact of COVID-19 on small-scale broiler production systems in Egypt and enhancing their preparedness and resilience to future pandemics, natural hazard risks, and market shocks.

## 1. Introduction

Poultry production in developing countries plays a crucial role in supporting livelihoods and fostering food security [1]. In particular, small-scale chicken raising is practiced by most rural households throughout developing countries for several reasons [2]. First, chickens lay eggs at the age of 6 months and are raised on short cycles. Second, chicken production systems require low levels of input and are efficient at converting feed into high-quality animal-source food [3]. Third, income generated from chicken raising is more stable and steadier than income from larger livestock [4]. Fourth, the fact that chickens are smaller and of lower value livestock makes them suitable livelihood options for vulnerable groups in the population who have few other options, particularly women and the poor [5]. Consequently, the potential contribution of poultry production systems, especially chicken systems, towards achieving the United Nations’ (UN) Sustainable Development Goals (SDGs) has been increasingly recognized by scholars and the development community [6,7].

The outbreak of the COVID-19 pandemic, caused by the novel coronavirus SARS-CoV-2 in early 2020, and the subsequent measures adopted by governments to contain the spread of the virus exerted a substantial impact on poultry production systems in developing countries [8,9]. As noted by Hobbs et al. [10], these containment measures have substantially affected the flow of agricultural commodities from producers to consumers and put food supply chains under the risk of disruption. In the same context, Hafez and Attia [11] revealed that the pandemic has disrupted many activities along poultry supply chains and posed unprecedented challenges to small poultry producers. There is growing evidence suggesting that the impact of the pandemic on poultry and other livestock supply chains threatens to worsen the livelihoods and food security status of poor and rural households in developing countries [12,13].

Unlike previous major disease outbreaks in recent decades (e.g., SARS, H7N9, Ebola, and Zika), the turbulence caused by COVID-19 mainly affected the downstream stages of the food supply chain [14,15]. That is, COVID-19 has not directly affected the production stages of the poultry supply chain [16]; however, disruptions in the downstream stages, such as transport and logistics as well as demand and consumption, have knock-back effects on producers [17,18]. For example, the RBN [19] found that COVID-19 restrictions adversely affected the ability of poultry producers in East African countries to access feed, water resources, and other production inputs and showed that exporters in these countries face significant reductions in demand for livestock-source foods in major importing markets. In Ethiopia, the spread of the virus decreased the demand for livestock-source foods due to mobility restrictions and the fear of consumers that fresh products may be contaminated with the virus [20,21]. Likewise, Lu et al. [22] showed that COVID-19 caused a shortage in farm labor on Indian farms due to restrictions on movement. In this context, small-scale poultry producers, in particular, struggled with several COVID-19 challenges, including disruptions in supply chains, a shortage of labor, and the malfunctioning of livestock markets, as well as price volatility and changes in consumer purchasing behavior [23,24]. Abu Hatab et al. [25] identified three major causes of the negative effects of the pandemic on small-scale poultry producers as compared to larger producers. First, small-scale producers and firms in these countries rely more heavily on labor than on machinery, and, thus, their poultry production has been especially compromised by containment measures and lockdowns. Second, small-scale producers in developing countries have limited logistical and financial capacity to implement hygiene measures, which, in turn, increases their vulnerability to COVID-19 effects. Third, the majority of small-scale agrifood-producing farms, including poultry farms, operate informally and, therefore, are excluded from the stimulus plans that governments have offered to private businesses [25]. 

While the impact of COVID-19 on food supply chains has prompted growing attention from both scholars and practitioners (see [9,26]), there is very little empirical evidence on the impact of the pandemic on poultry supply chains [27]. Specifically, a critical look into this emerging literature reveals three major shortcomings. First, the bulk of the literature is focused on developed countries, whereas there is still a lack of research based on the experiences of poultry producers in developing countries [18]. Second, most of the evidence is extremely aggregated and dominated by continental and regional-level studies and sector-level analyses, e.g., [28,29]. This neglects the fact that poultry systems in developing countries are heterogeneous and that their challenges in relation to the impact of COVID-19 vary according to geography, the influence of institutional frameworks, and the socioeconomic conditions in which they are located. Third, the literature is dominated by qualitative studies, e.g., [30], which tend to explore and describe the dynamics of COVID-19 and its effects on livestock chains in different contexts rather than measuring the extent of these effects. 

Against this background, this study contributes to the literature examining the impact of COVID-19 on small-scale livestock systems in developing countries by addressing a number of existing gaps in the understanding of its effects on small-scale poultry farms. Drawing on a survey of 205 small-scale commercial broiler farms (SCBFs) in Egypt, we first investigated the pathways through which the effects of the pandemic were transmitted to the SCBFs surveyed, and then we examined the determinants of the SCBFs’ perception of these effects. Our empirical analysis offers important insights into how COVID-19 has affected small-scale poultry producers in developing countries and how these producers can cope with the consequences of these effects. First, small producers still dominate poultry supply chains in developing countries; poultry systems represent a significant source of employment and contribute to household livelihood, food security, and nutrition. This implies that the negative effects of the pandemic may have substantial implications for achieving the Sustainable Development Goals in these countries [31]. Second, the projections that the occurrence of pandemics and disease outbreaks will be more frequent in the future [32] entail the necessity to understand the impact of the current pandemic on small-scale poultry farms in order to build up their preparedness, adaptation capacity, and resilience. Therefore, although this study is only limited to a single country (Egypt) and the empirical results are context-specific, there are potential lessons to be shared across developing countries with similar structures of broiler production systems. In particular, future research undertakings should expand this analysis to other contexts in developing countries to understand the channels through which the pandemic has affected small broiler producers and develop relevant and effective interventions to mitigate these effects. 

## 2. Brief Overview of the Poultry Sector in Egypt during the COVID-19 Pandemic

Over the course of the last few decades, poultry production in Egypt has experienced rapid development, driven by economic growth and increased demand for animal-source foods from the growing population, particularly urban middle-class consumers [33]. In 2018, total investments in this sector were calculated at around USD 4.5 billion, which created, directly and indirectly, 2.5 million employment opportunities along poultry value chains. Broiler production represents the largest element of Egypt’s poultry industry, with the production of broiler chickens in 2019 estimated at 1.8 billion birds, which cover 95% of the country’s total poultry consumption [34]. Broiler production systems, therefore, play a vital role in the food and agricultural economy of Egypt and make significant contributions to livelihoods and food security. 

Generally, broiler production systems in Egypt are classified into three main categories: industrial production systems, small-scale commercial production systems, and backyard production systems [33]. Of the country’s total broiler production, industrial and small-scale commercial systems supply around 90%, while household backyard poultry farms in rural areas and small towns supply the remaining 10%. According to statistics from the Egyptian Ministry of Agriculture and Land Reclamation (MALR), small-scale commercial systems represent around 70% of all poultry-producing farms in Egypt, almost 10% of the total value of agricultural production, and close to 25% of the total value of livestock production [35,36]. In this study, we focus on SCBFs, which produce less than 15,000 broilers per cycle [37]. Typically, SCBFs in Egypt are further subdivided based on the level of technology and the volume of production into farm-based and household-based systems. Most of SCBFs are simple units that produce between 4 and 5 cycles per year, with one shed of an average capacity of 5000 birds per cycle. Some farm-based broiler systems can have up to four sheds and a production capacity of 7 cycles per year, whereas household-based commercial broiler activities take place on the rooftops of rural houses, with significantly smaller numbers of birds and production cycles per year [35,38].

Like many other sectors of the Egyptian economy, the COVID-19 pandemic and the subsequent containment measures (e.g., mobility restrictions, stay-at-home orders, and limits to social contact) have drastically affected the poultry supply chains from farms to consumers [39,40]. In addition, consumer demand experienced dramatic declines due to public misperceptions linking poultry products to the transmission of COVID-19. As many SCBFs lack access to formal market channels, the closure of informal rural markets across the country led to a loss of income and market access. Furthermore, movement restrictions and blockages of transport routes during the pandemic were particularly obstructive for SCBFs because these measures disrupted access to production inputs (e.g., improved breeds, concentrate feeds, medicine, and vaccines), delivery, and marketing and, hence, led to the accumulation of production on the farms, leaving many SCBFs unable to sell their products [39]. Although the Egyptian government reacted to the economic impact of COVID-19 on small businesses by enacting fiscal and financial measures to alleviate liquidity constraints and facilitate continued production, many small businesses, including SCBFs, were excluded from these schemes due to a lack of official data about their activities [25]. In recognition of the role that SCBFs play in protein supply, as well as their significant contributions to employment, livelihoods, and food security, it is therefore important to understand the current impact of the pandemic on them in order to develop appropriate mitigation and adaptation strategies and build resilience to future pandemics. 

## 3. Materials and Methods

### 3.1. Study Area

**Figure 1:**
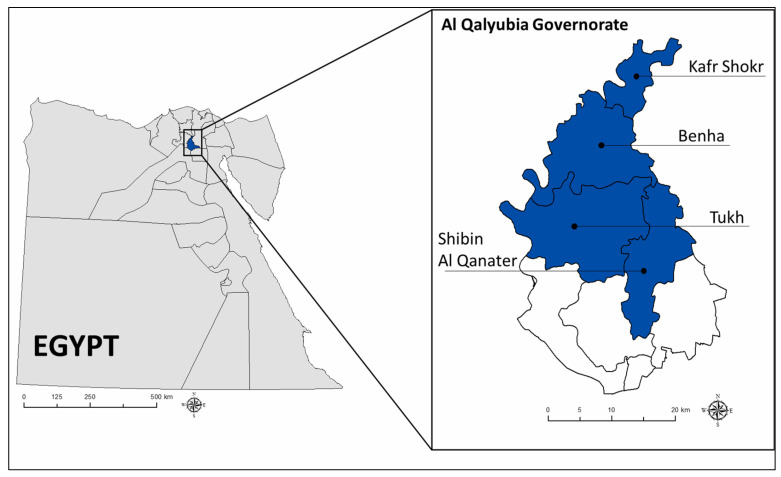
Study areas.

The field study was conducted in Al Qalyubia Governorate in Egypt. Situated at the apex of the Nile River delta, Al Qalyubia is ranked as the third-largest producer of broilers and the second-largest producer of layer birds in Egypt [41]. Between 2006 and 2018, the poultry industry in Qalyubia grew rapidly to become the leading agricultural economic activity. According to Fasina et al. [42], more than 90% of households in Al Qalyubia are engaged in poultry farming activities. Based on a focus group discussion carried out by the study team with a group of researchers and practitioners with experience in the poultry farming sector in Al Qalyubia, four study areas, as shown in Figure 1, were selected based on their specialization and relative importance to poultry production. These areas are Benha (42% of the farms surveyed), Kafr Shokr (31%), Tokh (19%), and Shibin Al Qanater (8%). 

### 3.2. Survey Design and Participants

Following the review of literature, presented in the previous section of this paper, on the impact of COVID-19 on small producers and enterprises in developing countries, e.g., [8,17,18,25], a structured questionnaire was developed in line with the objective of the study. The questionnaire was discussed with a selected small group of practitioners and researchers of broiler production who have experience in poultry farming in the selected areas. The final questionnaire was translated into Arabic after revisions and modifications (the original questionnaire was developed and written in English as the study team included non-Arabic speakers) and reviewed by two local researchers in the fields of agricultural economics and animal sciences in order to evaluate the face and content validity of the questionnaire and ensure question readability, clarity, and comprehensiveness. Then, a pilot study was performed on 15 randomly selected SCBFs before initiating data collection. The pilot samples were excluded from the final sample. Based on the results of the pilot study, minor amendments were made to the final questionnaire to ensure that the questions were clearly phrased and that respondents understood and responded as intended. 

The questionnaire contained informed consent, and it was structured into four main sections. The full questionnaire is available in the Supplementary Materials. Section 1 contains the demographics of the participants and general characteristics of the SCBFs surveyed. Section 2 consists of questions related to the perceived challenges caused by the COVID-19 pandemic on the SCBFs’ overall business performance. In Section 3, detailed questions were included to assess the perceived impact of the pandemic on various aspects of SCBF operations and performance. Finally, Section 4 contains questions related to the management strategies implemented by the farms surveyed in order to cope with the effects of the pandemic. The empirical analysis in this paper focuses on COVID-19 impacts on the surveyed firms; thus, no results related to Section 4 of the questionnaire (mitigation strategies) are reported in this paper. 

Face-to-face interviews were conducted between 30 September and 16 November 2020 with representatives of a randomly selected sample of small farms specializing in broiler farming in the study areas. Before the interviews, the participants were informed about the purpose of the study and the confidentiality of the information provided. All participants willingly consented to participation in the study. In addition, data were collected anonymously and analyzed using a coding system. During individual interviews, proper precautions and spatial distancing to protect enumerators and participants from the pandemic were maintained. In total, representatives of 205 SCBFs were interviewed for the study. As shown in Table 1, the interviewees consisted of farm owners (59%), managers (14%), agricultural engineers and veterinarians (24%), and other experienced workers (3%) who were perceived to possess the information, knowledge, and experience necessary to provide answers regarding the impact and challenges experienced by their respective farms during the pandemic. Of the 205 respondents, 174 (85%) were male, and 31 (15%) were female. Around half of the respondents were in the 25–44 age range, whereas the other half belonged to the age categories of 18–24 (18.5%) and 45 or older (30%). While considering the duration of broiler farming practice, nearly three-quarters recorded experience of less than 10 years, and a quarter recorded experiences equal to or above 10 years. About 46% of the respondents had gained university or postgraduate degrees, 25% had completed postsecondary technical education, 21.5% had completed primary or secondary education, and the remaining 7.5% were illiterate.

### 3.3. Outcome Measures and Covariates

The questionnaire contained 28 statements addressing the potential dimensions of COVID-19 effects on small-scale broiler producers in developing countries, which were identified in accordance with previous studies, e.g., [8,9,17,18,22,25]. Respondents were asked to indicate the extent to which each of these items (statements) represents a problem for their SCBFs on a five-point Likert scale. Specifically, the question was phrased as follows: “Thinking of your farm performance in 2019 and considering the current situation of COVID-19, how does each of the following sources of COVID-19 risks represent a problem for your farm business?” Response options consisted of “Minor problem”, “Moderate problem”, “Serious problem”, and “Very serious problem”. In order to minimize agreement bias due to item wording, we included two further response options: “Not at all a problem” and “Unable to judge”. None of the respondents indicated an inability to judge the effect of any of the items in this section of the questionnaire. The exact questions, the items included under each one, and the response options are presented in the attached questionnaire in the Supplementary Materials.

Variation in relation to the impact of COVID-19 on the SCBFs surveyed was examined according to key sociodemographic characteristics of the respondents (e.g., gender, age, experience, and the highest level of education) as well as SCBF characteristics and resource endowment (e.g., ownership structure, production system, main markets, number of employees, membership in producers’ organizations, value of total assets, and value of total sales in the past year).

### 3.4. Data Analysis

The statistical analysis of the data consisted of two steps. In the first step, a polychoric principal component analysis (polychoric PCA) [43,44] was conducted to reduce the number of variables/items included in the survey into a few interpretable combinations that capture the various effects of the pandemic on the farms surveyed. While the data in this study were categorical in nature, i.e., derived using the Likert rating scale, the literature suggests that standard principal component analysis (PCA) does not generate meaningful results on ordinal data [45]. Using polychoric correlations when ordinal data are used or in the presence of strong kurtosis or skewness is recommended, which is often the case of Likert items [46]. As noted by Gannon and Roberts [47], polychoric PCA generates consistent estimates of the proportion of explained variance; however, it is computationally intensive. Therefore, we used polychoric PCA, as suggested by Kolenikov and Angeles [44], which relies on polychoric and polyserial correlations that are estimated with maximum likelihood, with the assumption that there are latent normally distributed variables that underlie the ordinal categorical data. The polychoric correlations were estimated using the polychoricpca command in Stata 15, developed by Kolenikov and Angeles [43]. Similar to the standard PCA, the outcomes of a polychoric PCA reduce the input variables to principal components (PCs), which are of a magnitude less than the original dataset but preserve most of the information. Each component resulting from a polychoric PCA corresponds to a particular principal component that represents the underlying variables where there is most variance in the data. Thus, with the data grouped into components, examining these few new variables may develop a deeper understanding of the underlying causes that have led to the various effects of COVID-19, as perceived by the SCBFs surveyed. The identification of the number of components was based on the eigenvalue estimates generated from the Varimax-rotated factor analysis, where components with eigenvalues greater than unity were selected. The suitability of the data was assessed by computing the Kaiser–Meyer–Olkin (KMO) measurement of sampling adequacy and the Bartlett test of sphericity.

Next, the second step of data analysis was to investigate the determinants of the COVID-19 effects on the farms surveyed. Given the structure of the dataset and the categorical nature of the variables, an ordered-response model appeared to be the most appropriate approach [48]. When the order of response value is considered, the commonly-used model is an ordered logit model (OLM) regression [49]. The OLM combines the independent variables in order to estimate the probability that a particular event will occur, in this case, the probability that an SCBF would be affected by one of the dimensions of the pandemic-induced impact. More specifically, each of the principal components was used as a dependent variable in an OLM framework to examine the role of specified SCBF characteristics on the probability that the SCBFs were affected by each dimension of COVID-19 impact. More details on the use and application of OLMs can be found in McKelvey and Zavoina [50] and McCullagh [48]. Brief definitions of the explanatory variables included in the OLM estimates are provided in Table 2.

It should be, however, noted that, unlike linear regression models, regression coefficients of OLMs that are based on maximum likelihood procedures are difficult to interpret [51,52]. Consequently, we calculated the odds ratios (ORs) for the coefficients of the independent variables in order to facilitate the interpretation of the relevant size and magnitude of the effect of the explanatory variables on the probability that the SCBFs have been affected by each of the five dimensions of pandemic impact. The presentation and discussion of the OLMs will focus on the ORs obtained from the OLMs. In the context of our study, the ORs can be defined as the ratio of the odds of an effect of COVID-19 on the surveyed SCBFs occurring in one category of the independent variables to the odds of it occurring in another category. Thus, an OR of 1 indicates that the effect under study is equally likely in both categories. An OR greater than 1 indicates that the effect of the pandemic is more likely in a comparison category than the reference category.

## 4. Results and Discussion

### 4.1. Characteristics of the SCBFs Surveyed

As shown in Table 3, nearly two-thirds of the farms surveyed were individually owned, 28% were rented farms, and the remaining 7% were collectively owned (shared farms). Around 21% of the farms had more than one branch, with those having two or three branches equally representing around 9% of the sample, and those having 4 or more branches representing nearly 3% of the sample. With regard to the main broiler strain produced by the farms surveyed, the results showed that *Cobb* was the most dominant broiler strain, with the majority of the farms (83%) reporting this as the main broiler strain in their farms. The rest of the farms reported *Red Saso* (6.3%), *Ross* (5%), or other strains, including *Arbor Acres*, *Hubbard,* and *Plymouth Rock* (6%), as the main broiler strain in their farms. It should be noted that around 30% of the farms produced other strains in addition to the main strain and/or produced other poultry species (results not reported in Table 3). The number of production cycles per year varied among the survey farms, where about 57.5% of them raised between 5 and 7 batches, 18.5% raised less than 5 batches, and around 24% raised more than 7 batches.

With regard to farm labor, the results of Table 3 reveal that the majority of the farms surveyed (84%) employed less than five permanent workers, 13% employed between 6 and 10 workers, and very few farms (3%) employed more than 10 permanent workers. The structure of temporary farm labor was quite different; 61% of the farms hired between 1 and 5 temporary workers, around 9% hired between 6 and 10 temporary workers, and 30% operated without temporary labor. As per 2019, the SCBFs in our sample owned total assets varying from less than EGP 200 thousand (61.5%) to more than EGP 1 million (10%). Farms’ annual sales show a strong association with their sizes, reflected by the value of total sales. That is, 63% of the SCBFs surveyed showed total annual sales of less than EGP 300,000 in 2019, 14% showed annual sales between EGP 300,000–500,000, 13% had annual sales between EGP 500,000 to 1 million, and around 10% had annual sales of more than EGP 1 million.

### 4.2. Challenges Posed by COVID-19 Containment Measures to the SCBFs Surveyed

**Figure 2:**
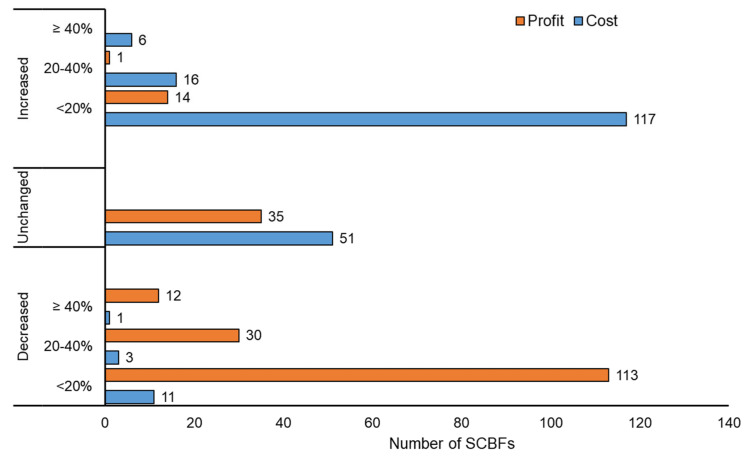
Changes in farm production costs and profitability in the first half of 2020 compared to the corresponding period in 2019 (*n =* 205).

In response to a question concerning the extent to which government containment measures affected the business performance of the SCBFs surveyed, around three-quarters (76%) of the farms indicated that these measures had exerted negative or very negative impacts on both the business environment of the poultry sector and their sales and profitability. A follow-up question was asked to understand the extent to which the pandemic containment measures affected their production and operation costs and farm profitability in the first half of 2020 compared to the corresponding period in 2019. As shown in Figure 2, about 68% of farms witnessed increases in the total cost of production and operation during the first six months of 2020, ranging from 20% (about 57% of the sample) to more than 40% (about 3% of the sample) compared to the corresponding period in 2019. In contrast, about 7% of the farms indicated that their total costs decreased or remained unchanged (about 25%) over the same period.

Figure 2 also indicates that changes in SCBFs’ costs of production between 2019 and 2020 had significant implications for farm profitability as nearly three-quarters of them indicated that their profitability had been reduced by a percentage of 20% (55% of the sample) to more than 40% (about 6% of the sample). The remaining quarter of SCBFs surveyed consisted of farms that witnessed no change in their profitability from broiler farming activities (17% of the sample) and those that had achieved higher profitability (only 7% of the sample). The specific pathways through which the COVID-19 pandemic influenced the costs, profits, and other aspects of farm operations, based on the results of the polychoric PCA, are discussed in the next section of this paper.

**Figure 3:**
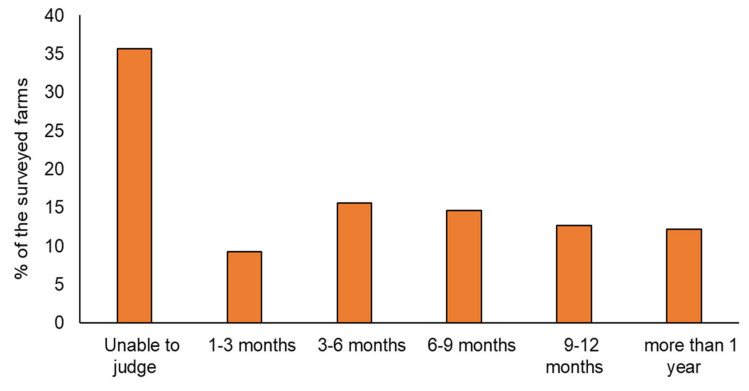
Anticipated duration for broiler farming business to return to normal, as anticipated by the farms surveyed (*n =* 205).

The respondents were also asked to indicate if they had temporarily shut down their broiler farming activities due to the pandemic. Responses revealed that 83 farms (about 41% of the sample) had been forced to close temporarily due to sluggish demand, falling prices, or to prevent the spread of the virus, causing unforeseen sales and staffing problems. A subsequent question concerning the number of weeks it took for them to reopen was asked of the farms that had shut down during the pandemic. Nearly 30% of the farms stated that they reopened within 1 to 4 weeks, with 37% reopening within periods ranging from 4 to 8 weeks. The remaining 27% resumed their broiler farming activities after relatively longer periods, ranging from 8 to 12 weeks or longer. With regard to respondents’ perception of when the broiler farming business would return to normal, around 10% of respondents estimated this period at 1–3 months, 16% at 3–6 months, 15% at 6–9 months, and 13% at 9–12 months (Figure 3). Another 12% of the surveyed farms were more pessimistic, believing that the poultry markets will take longer than a year to return to normal. Figure 3 also reveals that 36% of the SCBFs surveyed were uncertain and unable to make an assessment, which may be attributed to the fact that the COVID-19 pandemic presented unpreceded risks to the surveyed farms, making it hard for many of them to assess the trends and developments of the pandemic and its likely effects on broiler farming and poultry markets.

### 4.3. Main COVID-19 Impact Pathways on the SCBFs Surveyed

Tables S1 and S2 in the Supplementary Materials present the percent distribution and descriptive statistics of the original variables (statements) that were used in the polychoric PCA to assess the perception of SCBFs of various effects of the COVID-19 pandemic. As shown in Table 4, the polychoric PCA yielded five factors with eigenvalue estimates greater than unity, which collectively accounted for about 86% of the variance in the original variables. The computed KMO measure of sampling adequacy indicated that around 65.7% of the variance in our variables is caused by underlying factors, implying the suitability of polychoric PCA for our data. The results of the Bartlett test of sphericity showed that the chi-square test statistic was 3302.24, with a significance level of *p* < 0.0000, confirming reliability and suitability for examining the effects of COVID-19 on the SCBFs in the sample. Figure S1 in the Supplementary Materials shows the scree plot of eigenvalues after polychoric principal component analysis.

The first category of impact, denoted as “*availability of production inputs*”, had a scale reliability coefficient of 0.901 and consisted of the COVID-19 impact related to the shortage of adequate feed, vaccines, and veterinary medicines, the reduced availability of equipment used for collecting litter, and the short supply of chicks during the peak months of the COVID-19 pandemic. As noted by Biswal et al. [30], the COVID-19 pandemic has disrupted many activities along the supply chain and led to the lack of availability of feed and other inputs such as veterinary supplies and vaccines as well as other broiler farming inputs that are essential to sustain farm operations [27]. These disruptions, according to Ejeromedoghene et al. [53], threaten to reduce livestock production, disrupt market activities, and raise the price of poultry products in developing countries.

The second set of impacts, labeled “*production operational costs*”, showed a scale reliability coefficient of 0.759. This category contains the impact related to the increased cost of chicks, feeding, and vaccines and veterinary medicines. Furthermore, it included impacts associated with increased mortality rates and increased cost of transportation. These findings are in keeping with the findings of Amjath-Babu et al. [54] and Seleiman et al. [55], showing that mobility restrictions and other pandemic containment measures have substantially increased the cost of many operations along the agricultural supply chain. In this respect, Mujeri et al. [56] illustrated that the pandemic decreased the economic activities of small enterprises and caused operational difficulties, including decreased labor productivity and increased costs of transportation. More so, a survey of the impact of COVID-19 on Egyptian SMEs [40] revealed that 3% of the enterprises (*n =* 283) permanently ceased business activities, around half of them stopped temporarily, and close to two-thirds faced a projected 24% to 50% increase in operating costs and up to a 39% decrease in revenue.

The third category was denoted “*labor and human resources*”, and it had a scale reliability coefficient of 0.749. The farms studied illustrated that the pandemic had substantial effects on their human resources as their employees were unable to commute to work, which resulted in high rates of worker absenteeism. Worker productivity was adversely affected by the reduction in the number of working days, the reduced working hours, and worker absenteeism due to the fear of being infected [30]. Furthermore, several farms indicated that they had to lay off a number of workers during the peak period of the pandemic and, accordingly, lost skilled labor because of the scaling down of production activities and their inability to pay wages. A number of reports have shown that the pandemic created profound challenges for farm labor in Egypt, e.g., [57,58], which may be explained by the fact that SCBFs in developing countries rely more heavily on human labor than on machinery for their business operations [22]. Thus, the continuity of SCBFs’ activities was compromised by restrictions on human mobility and other containment measures [25].

The fourth category, labeled “*market demand and sales*”, had a scale reliability coefficient of 0.715. Price volatility and market fluctuations were the major effects in this category, together with market access issues and difficulty in supplying products to local markets. Furthermore, this category includes effects related to falling demand by the food service industry, which includes restaurants and other types of prepared-food retailers who consume poultry products. These findings are in concert with the findings of Cowling et al. [59], who showed that SMEs are very vulnerable to unexpected events that significantly decrease household consumption and reduce the market demand, sales, and revenue of these enterprises. Similarly, the closure of schools, universities, and restaurants during the pandemic, together with the night-time curfew and other restrictions on human mobility as well as the loss of income in several segments of the population due to layoffs, reduced consumer purchasing power and the demand for poultry products [30,54].

The fifth category, labeled “*farm finances*”, with a scale reliability coefficient of 0.619, contains effects related to financial flows and access to credit. Because of the measures taken by the government to control the spread of COVID-19, several farms reported that their farm businesses were seriously impacted financially. As a result, these farms reported difficulties in paying rent and loans. Furthermore, the pandemic complicated their access to capital and financial services, and many of them had to seek new loans to be able to cover their operational costs. In their quest to obtain credit, SCBFs encountered two challenges, namely, the rising interest rates, which may hamper their ability to make repayments, and the reluctance of many financial institutions to provide loans to small businesses during the pandemic. This finding is in accord with the findings of La Rocca et al. [60], indicating that unexpected events amplify the financial challenges that small businesses generally face in normal times and leave them with little cash flow to cover recurrent expenses, including wages, bank interest on loans, and rent of premises. In this respect, Fan et al. [61] and Hafez and Attia [11] revealed that the pandemic has worsened the economics of poultry farming and that most small-scale producers are on the brink of bankruptcy, experiencing capital shortages and fearing that they will not be able to continue their work due to interruptions in the livestock supply chain.

### 4.4. Determinants of COVID-19 Impact on the SCBFs Surveyed

Table 5 presents the ORs obtained from the estimated OLMs presented. Table S3 in the Supplementary Materials presents descriptive statistics of the dependent variables used in the model estimations, and Table S4 in the Supplementary Materials reports the estimation results of the five OLM models.

In terms of the personal characteristics of SCBF operators, the results in Table 5 reveal that compared to their male counterparts, female-led SCBFs were about 2.21, 1.88, and 1.49 times more likely to perceive COVID-19 impacts related to production and operational costs, farm finances, and availability of production inputs, respectively. This finding that female-led SCBFs perceived more financial challenges is in keeping with extensive and still growing literature showing that small enterprises run by women encounter significant financial obstacles (e.g., lack of access to credit and financial services) that impede female entrepreneurship and prevent women from participating in agricultural and livestock supply chains in developing countries [62,63]. Studies that examined gender differences in access to financial services in developing countries have found that women possess lower financial literacy than men [64] and that women-led enterprises encounter greater financial obstacles and are more likely to rely on informal financing (see [65,66]). Furthermore, the results in Table 5 show that female-run SCBFs are more likely to perceive COVID-19 impacts in relation to the availability of production inputs and costs of production and operation. In this respect, the ILO [67] has shown that Egyptian women entrepreneurs not only face greater financial constraints but also face significant production and operational costs that reduce their competitiveness and growth opportunities. Raghuvanshi et al. [68] and Kathuku [69] identified a number of barriers that increase production and operational costs in female-run enterprises in developing countries, including the smaller scale of their business operations, their lack of membership of agribusiness organizations and networks, their lack of access to information on inputs and markets, and their lack of business contacts and networks.

Although many of the ORs related to the educational attainment of SCBF operators were statistically insignificant in all the estimated models, the significant estimates tend to suggest that farms operated by farmers with a secondary level of education or above were less likely to perceive COVID-19 impacts on production and operational costs and, to a lesser extent, on input availability, human resources, and farm finances. This finding is consistent with prior research that suggests that entrepreneurs with higher levels of education and training have greater capability to adapt their enterprise to changes in the business environment and that technical and managerial skills enable small businesses to manage and recover from external business shocks [70,71]. In the same vein, the results indicate that the experience of SCBF operators in broiler farming (number of years in Table 5) tends to be associated with less COVID-19 impact. In particular, SCBFs operated by managers with experience of between 5 and 10 years or more than 15 years in broiler farming were 0.91 and 0.72 times less likely than those with experience of less than 5 years in the business to perceive COVID-19 impacts on labor and human resources. Moreover, firms operated by managers with experience between 5 and 10 years were significantly less likely than those with less than 5 years’ experience to perceive impacts on costs of production and operation, labor and human resources, and sales and consumer demand. This finding is consistent with findings of prior research on small business perceptions of the impact of nature-induced and socioeconomic risks, where managerial experience is a significant determinant of risk perception and positively associated with risk management decisions [72,73].

In relation to SCBF characteristics, ownership structure was found to be a significant determinant of COVID-19 impact on SCBFs. That is, rented farms were significantly more likely to perceive impact related to farm finances (OR = 3.08), sales and consumer demand (OR = 1.97), production and operational cost (OR = 1.72), and difficulties in sourcing production inputs (OR = 1.46). Likewise, the results revealed that collectively owned farms (shared) were more likely than individually-owned SCBFs to perceive COVID-19 impacts related to sourcing production inputs (OR = 7.13) and labor and human resources (OR = 2.46). In this respect, Mishra et al. [74] illustrated that the type of business organization may have an impact on the financial performance of farms. Moreover, Abd El-Hamed et al. [75] illustrated that the better performance of individually owned poultry farms in Egypt may be attributed to the fact that these farms manage resources efficiently and have much simpler and more direct decision-making processes compared to other forms of farm ownership. Furthermore, individuals running this type of farm have an incentive to perform well as the returns accrue directly to them. With regard to broiler production systems, the results indicated that farm-based systems were 2.38 and 2.34 times more likely than household-based systems to perceive COVID-19 impacts on labor and human resources and on the availability of production inputs. On the contrary, household-based systems were found to be significantly less likely than farm-based systems to perceive the impact related to farm production and operational costs. This could be attributed to the characteristics of farms belonging to this category, including their relatively smaller size, their reliance on family labor, and selling directly to consumers without incurring transport costs [33,76].

Concerning SCBF labor, the results revealed that compared to SCBFs employing less than 5 permanent workers, farms employing between 6 and less than 10 permanent workers were 1.94, 1.67, and 1.43 times more likely to perceive COVID-19 impacts related to the farm’s labor and human resources, farm finances, and production and operational costs, respectively. The results related to temporary workers further confirm this finding, where SCBFs hiring between 6 and 10 temporary workers were more likely to perceive COVID-19 impacts related to production and operational costs and human resources. These findings are expected, given that the categories of “operational cost” and “labor and human resources” of COVID-19 impact consist mainly of components related to SCBF labor, such as the increased cost of wages, the layoff of workers and loss of skilled labor, and high rates of worker absenteeism (see Table 4). Furthermore, these findings support those of Hashem et al. [17], which pointed out that livestock production is a heavily labor-dependent sector, and thus, the outbreak of COVID-19 caused severe shortages in the workforce and professional services, leading to substantial disruptions in the livestock supply chain, including a decrease in the economic and productive efficiency of livestock farms.

In relation to the value of SCBF total assets, the results indicated that farms with higher values of total assets perceived significantly less COVID-19 impact related to the availability of production inputs in particular. One explanation for this finding is that larger-sized SCBFs with higher values of total assets are customarily more integrated and have transportation means and storage facilities to transport and store production inputs. In this regard, the FAO [37] has illustrated that small broiler farms have poor transport links and lack storage facilities, which disadvantage them and make them less flexible in adjusting to outbreaks and unexpected shocks. The results also showed that higher values of total assets are associated with less perceived financial effects (OR = 0.335 for SCBFs with total assets between EGP 500,000–1,000,000). As shown by Abu Hatab et al. [25], total assets of small and medium-sized enterprises function as a buffer against unexpected shocks of the inherently risky agribusiness in Egypt, and thus, higher total assets may reduce the likelihood that SCBFs will experience challenges related to operational and recurrent expenses, including wages, interest on loans, and rent. Noteworthy, most of the ORs related to the “total sales” variable were statistically insignificant. A similar finding was reported by Abu Hatab et al. [25] in Egyptian agrifood SMEs and was explained by the tendency of these enterprises in Egypt to underestimate their turnover and total assets.

Interestingly, the results revealed that the SCBFs that market their products in local markets only are 2.56 and 1.61 times more likely to perceive COVID-19 effects in relation to consumer demand and farm finances, respectively, compare to SCBFs marketing their products in both the domestic and provincial markets of other Egyptian governorates. This finding is in line with the findings of Srinivas et al. [77] and Moraine et al. [78], showing that diversified markets of livestock systems decrease farm vulnerability to external shocks and market instability. In connection with this finding, the results indicated that SCBFs without membership of poultry producer organizations are significantly more likely to perceived negative COVID-19 effects in relation to the availability of production inputs (OR = 1.74) and consumer demand (OR = 1.25). In this respect, several studies have shown that membership of poultry farmer organizations increases farm efficiency and risk perception through the sharing of ideas and experiences among members and the technical support and marketing services that these organizations provide to members [79,80].

## 5. Conclusions and Implications for Mitigation Measures

This study aims to add to the literature on COVID-19 impacts on small-scale livestock systems in developing countries by investigating the effects of the pandemic on a sample of 205 small-scale commercial broiler farms (SCBFs) in Egypt. Overall, the empirical results revealed that COVID-19 has adversely affected SCBFs’ supply chains in substantial ways depending on their resource endowment and production and management characteristics. Our findings underscore several *general* and *specific* implications for formulating effective strategies for mitigating the effects of COVID-19 and future pandemics on small-scale broiler systems in developing countries.

In *general* terms, our findings call for the need for the sustainable transformation of small-scale broiler systems in developing countries through the adoption of more integrated resilience-based approaches to take on effective preventive measures before disruptions and recovery measures after their occurrence. This requires a comprehensive assessment of various types of SCBF vulnerabilities and an understanding of what the magnitude and breadth of impact will be when these vulnerabilities are exposed. In this respect, the substantial negative impact that COVID-19 exerted on small-scale broiler systems, as evidenced by our empirical results, should be leveraged as an opportunity to “build back better” and enhance SCBFs’ preparedness and adaptation to various threats. That is, developing countries should utilize the lessons learned from COVID-19 impacts as well as the rehabilitation activities of past zoonotic disease outbreaks to strengthen small-scale broiler supply chains at various stages and enhance their preparedness and response plans in the face of emerging disruptions.

In addition, our results generally underscore the need for holistic management approaches that recognize the complexity of small-scale livestock systems in developing countries and the ways they behave and respond to different shocks and disruptions. In this respect, our results imply that although the adoption of strict and immediate containment measures was essential for controlling the virus and protecting public health, policy responses to COVID-19 must consider the likely effects on small businesses such as SCBFs since disruptions to such socioeconomically important supply chains will intensify human suffering from the pandemic. Thus, the adoption of more holistic management approaches would offer an opportunity to take a system perspective on the impacts of pandemics on all stages of small-scale broiler chains, from input supply and production to consumption, and allow for comprehensive analyses of their influence on broiler supply chains and consumer demand. In this context, coalescing diverse actors and stakeholders at all levels around One Health approaches is crucial, as these approaches integrate animal, human, and environmental health and place food systems at their center.

In more *specific* terms, our results imply that comprehensive and robust financial support to SCBFs should be a key element in mitigation strategies against supply chain disruptions since financial measures would be crucial for their survival and recovery. Therefore, it is important to promote and facilitate SCBFs’ access to finance by providing short-term stimulus packages to support sales and cash flow. In addition, our results revealed that female-led SCBFs are more likely to perceive negative impacts from unexpected events. This highlights a major threat to the empowerment of women in poultry value chains, which may have negative repercussions not only for female entrepreneurs and individuals but also for the entire value chain. Therefore, there is a need to develop effective approaches to support female broiler entrepreneurs during times of supply chain disruptions by offering funding programs with subsidized interest rates and providing tailored technical support and training and advisory services. Furthermore, the results suggest that building the capacity of SCBFs operators is necessary to equip them with the skills and abilities to deal with various business risks. In particular, SCBF operators and workers should be trained in risk projection and preparedness, management techniques, and risk management strategies. It is also essential to build the capacity of broiler producer associations to enhance effective communication, the sharing of good practices, and strategic partnerships with government and stakeholders in order to cope with the effects of the pandemic. Finally, the results indicate that improving small producers’ access to markets is an important area of investment since producer access to markets is dependent on infrastructure and the prices in these markets. Thus, improving infrastructure in rural areas would exert a favorable effect on diversifying and improving SCBFs’ access to markets and, thus, reduce their vulnerability to extreme events and market shocks.

## Figures and Tables

**Table 1:** Sociodemographic characteristics of respondents (*n =* 205).

Variable	Frequency	Percentage (%)
**Gender**		
Male	174	84.88
Female	31	15.12
**Role in the farm**		
Owner	121	59.02
Farm manager	29	14.15
Agricultural engineer	31	15.12
Veterinarian	18	8.78
Other (e.g., worker)	6	2.93
**Age**		
18–24	38	18.54
25–34	51	24.88
35–44	56	27.32
45–54	38	18.54
Over 55	22	10.73
**Education**		
Illiterate	15	7.32
Primary	17	8.29
Secondary	27	13.17
Technical	52	25.37
University or above	94	45.85
**Experience**		
<5	91	44.39
5–10	61	29.76
10–15	31	15.12
>15	22	10.73

Source: survey results.

**Table 2:** Definition of variables used in the estimation of the ordered logit models.

Variable	Type	Definition
Gender	binary	1 = male; 0 = female
Education	poly	1 = illiterate; 2 = primary; 3 = secondary; 4 = technical; 5 = university or above
Ownership structure	poly	1 = individually-owned farms; 2 = rented farms; 3 = shared farms
Number of years in broiler farming	poly	Experience of broiler farming of SCBF operators. 1 = <5; 2 = 5–10; 3 = 10–15; 4 = >15
Production system	binary	1 = household-based systems; 2 = Farm-based broiler systems
Number of permanent workers	poly	1 = 1–5 workers; 2 = 6–10 workers; 3 = ≥10 workers
Number of temporary workers	poly	1 = no temporary workers; 2 = 1–5 workers; 3 = 6–10 workers
Total assets	poly	1 = <200,000; 2 = 200,000–500,000; 3 = 500,000–1,000,000; 4 = ≥1,000,000
Annual sales	poly	1 = <300,000; 2 = 300,000–500,000; 3 = 500,000–1,000,000; 4 = ≥1,000,000

**Table 3:** Characteristics of the surveyed small-scale commercial broiler farms (*n =* 205).

Characteristics	Frequency	Percentage (%)
**Production system**		
Household-based systems	64	31.22
Farm-based systems	141	68.87
**Ownership structure**		
Individually owned farms	132	64.39
Rented farms	58	28.29
Shared farms	15	7.32
**Number of branches**		
1	162	79.02
2	19	9.27
3	18	8.78
≥4	6	2.93
**Number of cycles/farm/year**		
<3	13	6.34
3–5	25	12.19
5–7	118	57.56
≥7	49	23.9
**Broiler strains**		
Cobb	170	82.93
Red Saso	13	6.34
Ross	10	4.88
Other	12	5.86
**Number of permanent workers**		
1–5 workers	172	83.9
6–10 workers	27	13.17
≥10 workers	6	2.93
**Number of temporary workers**		
No. of temporary workers	62	30.24
1–5 workers	125	60.98
6–10 workers	18	8.78
Total assets (EGP^*^)		
<200,000	126	61.46
200,000–500,000	36	17.56
500,000–1,000,000	22	10.73
≥1,000,000	21	10.24
**Annual sales (EGP)**		
<300,000	130	63.42
300,000–500,000	29	14.15
500, 000–1,000,000	26	12.68
≥1,000,000	20	9.76

US dollar = 15.754 Egyptian pound (EGP) on 1 October 2020. Source: survey results.

**Table 4:** Polychoric PCA for the main impact pathways of the pandemic on the farms surveyed.

Items	PC1	PC2	PC3	PC4	PC5
Decreased value of total sales compared to 2019				0.788	
Difficulty in access to markets				0.680	
Volatility in market prices				0.630	
Falling market demand (retailers and consumers)				0.593	
Reduced availability of feed	0.913				
Reduced availability of vaccines and veterinary medicines	0.974				
Reduced availability of equipment used for collecting litter	0.785				
Short supply of chicks	0.803				
Lack of availability of adequate feed	0.721				
High rates of worker absenteeism			0.858		
Layoff of workers and loss of skilled labor			0.822		
Inability to pay back farm loans					0.616
Higher interest rate on new loans					0.623
Limited capital and lack of access to finance					0.495
Increased cost of chicks		0.753			
Increased cost of feed		0.797			
Increased cost of vaccines and veterinary medicines		0.744			
Decreased worker productivity			0.497		
Inability to pay farm rent			0.464		
Increased cost of wages			0.552		
Increased mortality rates		0.473			
Increased transportation cost		0.510			

PC1 = input availability; PC2 = production and operational costs; PC3 = labor and human resources; PC4 = consumer demand and firm sales; PC5 = farm finances. Rotation method: Varimax with Kaiser normalization. Values >0.4 are reported.

**Table 5:** Calculated odds ratio of the explanatory variables in the ordered logit models examining the determinants of COVID-19 impacts on the farms surveyed.

Independent Variables	Model 1	Model 2	Model 3	Model 4	Model 5	
**Gender** (Ref = male)	1.492 * (0.405)	2.212 ** (0.845)	1.534 (0.610)	0.704 (0.274)	1.888 ** (0.708)	
**Education** (Ref = illiterate)						
Primary	0.428 (0.285)	0.140 *** (0.091)	1.266 (0.801)	0.571 (0.361)	0.601 (0.386)	
Secondary	0.864 ** (0.361)	0.484 ** (2.691)	0.284 ** (0.173)	0.771 (0.468)	0.191 *** (0.118)	
Technical	0.594 (0.332)	0.354 * (0.192)	1.033 (0.553)	0.652 (0.363)	0.553 (0.294)	
University or higher	0.731 (0.399)	0.201 *** (0.105)	1.827 (0.943)	0.742 (0.403)	0.437 (0.227)	
**Number of years** (Ref = <5 years)						
5–10 years	0.597 (0.188)	0.493 ** (0.153)	0.692 *** (0.157)	0.586 * (0.182)	1.025 (0.318)	
10–15 years	1.567 (0.624)	0.667 (0.272)	0.912 ** (0.429)	0.810 (0.332)	0.798 (0.323)	
>15 years	0.726 (0.364)	0.635 (0.322)	0.722 *** (1.875)	0.622 (0.334)	1.026 (0.479)	
**Ownership** (Ref = individually owned)						
Rented	1.467 ** (0.147)	1.727 * (0.532)	1.227 (0.386)	1.974 ** (0.593)	3.083 *** (0.959)	
Shared	7.133 *** (3.883)	1.588 (0.894)	2.463 * (1.347)	0.620 (0.313)	2.115 (1.186)	
**Production system** (Ref = farm-based)	2.343 *** (0.760)	0.468 ** (0.149)	2.381 *** (0.727)	1.085 (0.330)	0.762 (0.236)	
**Number of permanent workers** (Ref = 1–5 workers)						
6–10 workers	1.246 (0.590)	1.435 ** (0.570)	1.945 ** (0.811)	0.371 ** (0.183)	1.675 ** (0.662)	
>10 workers	0.528 (4.511)	1.702 (1.565)	4.273 (3.775)	0.239 * (0.200)	0.512 (0.461)	
**Number of temporary workers** (Ref = no. of temporary workers)						
1–5 workers	0.924 (0.506)	1.411 (0.828)	1.767 (0.964)	1.653 (0.926)	0.996 (0.521)	
6–10 workers	0.978 (0.325)	2.075 ** (0.662)	0.413 *** (0.129)	1.516 (0.472)	1.457 (0.459)	
**Total assets** (Ref = < 100,000 EGP)						
100,000–200,000	0.370 ** (0.180)	1.627 (0.761)	0.859 (0.436)	0.728 (0.363)	1.042 (0.490)	
200,000–500,000	0.135 *** (0.087)	1.510 (0.983)	0.172 (0.118)	0.720 (0.483)	1.177 (0.769)	
500,000–1,000,000	0.062 *** (0.061)	4.312 (4.166)	0.851 (0.815)	1.772 (1.679)	0.335 ** (0.100)	
**Annual sales** (Ref = < 100,000 EGP)						
100,000–300,000	0.652 (0.347)	0.633 (0.344)	0.665 (0.332)	0.434 (0.238)	1.984 (1.146)	
300,000–500,000	1.009 (0.679)	1.887 (1.390)	0.569 (0.386)	0.598 (0.412)	1.718 (1.178)	
500,000–1,000,000	2.551 (2.872)	1.473 (1.517)	1.580 (1.608)	0.667 (0.691)	1.198 (1.165)	
**Markets** (Ref = local and provincial markets)	0.851 (0.372)	1.161 (0.532)	1.327 (0.577)	2.562 ** (1.094)	1.613 * (0.447)	
**Membership in producer organizations** (Ref = members)	1.258 ** (0.350)	0.561 (0.252)	1.330 (0.598)	1.742 ** (0.568)	1.344 (0.560)	

Calculated odds ratios and standard errors (in parentheses) are reported. Dependent variables are *input availability* (Model 1), *production and operational costs* (Model 2), *labor and human resources* (Model 3), *consumer demand and firm sales* (Model 4), and *farm finances* (Model 5). Statistically significant at * 10%, ** 5%, and *** 1% levels.

## Data Availability

The data presented in this study are available on request from the corresponding authors.

## Supplementary Materials

The following are available online at https://www.mdpi.com/article/10.3390/ani11051354/s1, Figure S1: Scree plot of eigenvalues after polychoric principal component analysis, Table S1: Percent distribution of SCBFs’ perception of various effects of the COVID-19 pandemic, Table S2: Descriptive statistics of the statements included in the polychoric principal component analysis, Table S3: Pairwise correlation between principal components of pandemic effects, Table S4: Descriptive statistics of dependent variables used in the estimation of the ordered logit models analysis, Table S5: Estimated results of OLMs examining factors determining Covid-19 impacts on the SBFS surveyed, Questionnaire: “Determinants of SARS-CoV-2 impact on small-scale commercial broiler production systems in Egypt: Implications for mitigation strategies’’.
