# Supplementary material for: Determinants of SARS-CoV-2 Impacts on Small-Scale Commercial Broiler Production Systems in Egypt: Implications for Mitigation Strategies
Source: Animals (Basel). 2021 May 10;11(5):1354. doi: 10.3390/ani11051354 (PMC8151507; doi:10.3390/ani11051354)
Supplement: Supplementary file 1 [file animals-11-01354-s001.zip › animals-1199668-supplementary.pdf]

Supplementary material to manuscript

Determinants of SARS-CoV-2 impact on small-scale commercial broiler production systems in Egypt: Implications for mitigation strategies

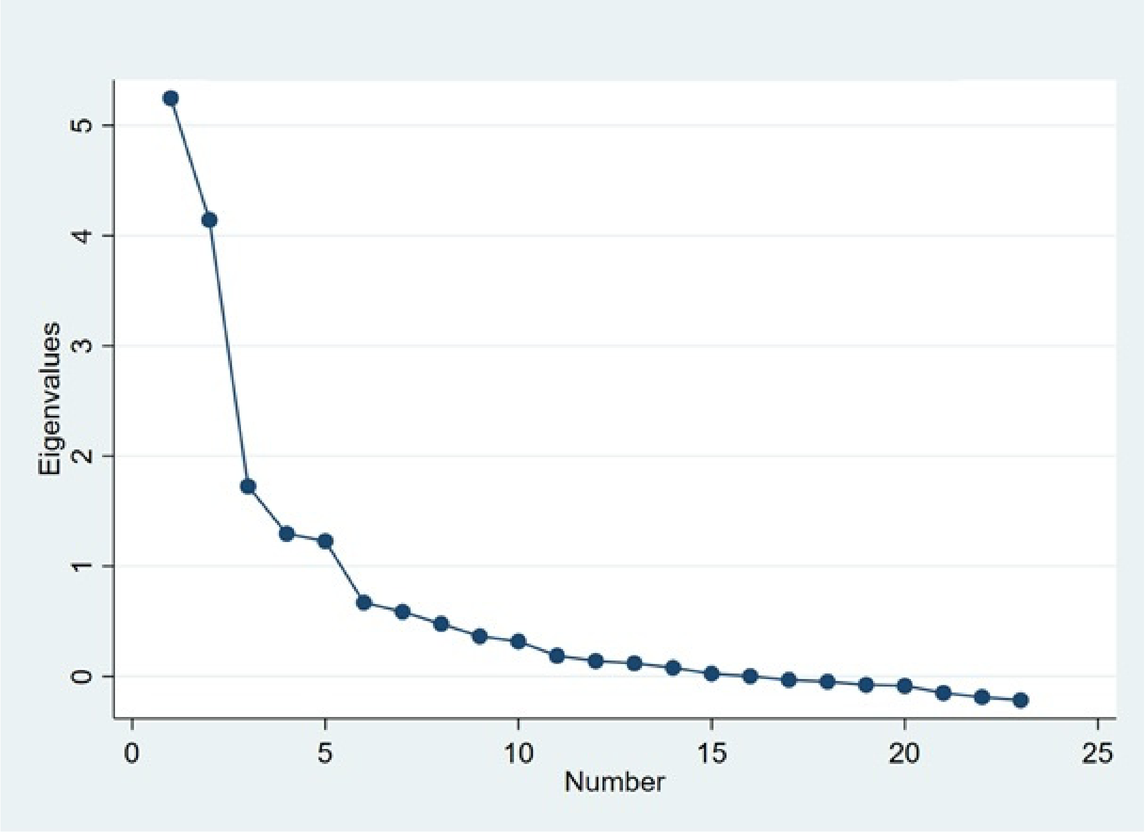

Figure S1. Scree plot of eigenvalues after Polychoric principal component analysis

Table S1. Percent distribution of SCBFs' perception of various effects of the COVID-19 pandemic

COVID-19 impact	*SCBFs' perception of the COVID-19 impact*										Total	
	Very negative		Negative		No effect		Positive		Very positive			
	Freq.	%	Freq.	%	Freq.	%	Freq.	%	Freq.	%	Freq.	%
Decreased value of total sales compared to 2019	9	4.4	19	9.3	23	11.2	72	35.1	82	40	205	100
Difficulty in access to- and supplying local products	14	6.8	21	10.2	29	14.1	66	32.2	75	36.6	205	100
Volatility in market prices	6	2.9	29	14.1	42	20.5	54	26.3	74	36.1	205	100
Falling market demand (retailers and consumers)	17	8.3	32	15.6	51	24.9	66	32.2	39	19.0	205	100
Reduced availability of feed	31	15.1	66	32.2	101	49.3	3	1.5	4	2.0	205	100
Reduced availability of vaccines and veterinary medicines	30	14.6	58	28.3	111	54.1	2	1.0	4	2.0	205	100
Reduced availability of equipment used for collecting litter	20	9.8	22	10.7	35	17.1	63	30.7	65	31.7	205	100
Short supply of chicks	30	14.6	61	29.8	104	50.7	5	2.4	5	2.4	205	100
Lack of availability of adequate feed	42	20.5	64	31.2	86	42.0	9	4.4	4	2.0	205	100
High rates of worker absenteeism	47	22.9	50	24.4	72	35.1	28	13.7	8	3.9	205	100
Layoff of workers and loss of skilled labor	53	25.9	47	22.9	75	36.6	19	9.3	11	5.4	205	100
Inability to pay back farm loans	15	7.3	22	10.7	58	28.3	61	29.8	49	23.9	205	100
Higher interest rate on new loans	18	8.8	11	5.4	125	61.0	31	15.1	20	9.8	205	100
Limited capital and lack of access to finance	12	5.9	14	6.8	79	38.5	54	26.3	46	22.4	205	100
Increased cost of chicks	1	0.5	8	3.9	36	17.6	93	45.4	67	32.7	205	100
Increased cost of feed	0	0.0	5	2.4	33	16.1	98	47.8	69	33.7	205	100
Increased cost of vaccines and veterinary medicines	0	0.0	4	2.0	110	53.7	62	30.2	29	14.1	205	100
Decreased worker productivity	0	0.0	0	0.0	38	18.5	84	41.0	83	40.5	205	100
Inability to pay farm rent and hire seasonal labor	0	0.0	5	2.4	123	60.0	61	29.8	16	7.8	205	100
Increased cost of wages	0	0.0	4	2.0	138	67.3	40	19.5	23	11.2	205	100
Increased cost energy (e.g. electricity and heating)	0	0.0	0	0.0	26	12.7	64	31.2	115	56.1	205	100
Increased mortality rates	0	0.0	10	4.9	148	72.2	29	14.1	18	8.8	205	100
Increased transportation cost	0	0.0	3	1.463415	65	31.7	82	40.0	55	26.8	205	100

Note: the question on the survey was: "*Thinking of your farm's overall performance in 2019 and considering the current situation of COVID-19, how you would assess the impact of the pandemic in relation to the following aspects of your business*?" None of the 205 respondents chose the option "unable to judge", thus all answers ranged between "very negative" to "very positive".

Table S2. Descriptive statistics of the statements included in Polychoric principal component analysis

Variable	Obs	Mean	Std. Dev.	Min	Max
Decreased value of total sales compared to 2019	205	3.971	1.133	1	5
Difficulty in access to- and supplying local products	205	3.815	1.227	1	5
Volatility in market prices	205	3.785	1.164	1	5
Falling market demand (retailers and consumers)	205	3.38	1.197	1	5
Reduced availability of feed	205	2.429	.835	1	5
Reduced availability of vaccines and veterinary medicines	205	2.473	.826	1	5
Reduced availability of equipment used for collecting litter	205	2.449	.693	1	5
Short supply of chicks	205	2.483	.861	1	5
Lack of availability of adequate feed	205	2.361	.922	1	5
High rates of worker absenteeism	205	2.512	1.105	1	5
Layoff of workers and loss of skilled labor	205	2.454	1.131	1	5
Inability to pay back farm loans	205	3.522	1.178	1	5
Higher interest rate on new loans	205	3.117	.968	1	5
Limited capital and lack of access to finance	205	3.527	1.092	1	5
Increased cost of chicks	205	4.059	.838	1	5
Increased cost of feed	205	4.127	.763	2	5
Increased cost of vaccines and veterinary medicines	205	4.22	.738	3	5
Decreased worker productivity	205	3.566	.755	2	5
Inability to pay farm rent and hire seasonal labor	205	3.429	.672	2	5
Increased cost of wages	205	3.4	.711	2	5
Increased cost energy (e.g. electricity and heating)	205	4.434	.708	3	5
Increased mortality rates	205	3.268	.687	2	5
Increased transportation cost	205	3.922	.801	2	5

Table S3. Descriptive statistics of dependent variables used in the estimation of the ordered logit models analysis

Variable*	Obs.	Mean	Std. Dev.	Min	Max
Input availability	205	3.116	.898	1.148	5.742
Cost	205	5.003	.74	2.944	7.332
Labor	205	2.394	.925	.786	4.729
Sales	205	3.018	1.07	-.134	5.088
Finances	205	2.867	.937	-.041	4.747

* Note: these dependent variables are based on the five principal components generated by the Polychoric principal component

Table S4. Estimated results of OLMs examining factors determining Covid-19 impacts on the SBFS surveyed

Independent Variables	Model 1	Model 2	Model 3	Model 4	Model 5
**Gender** (Ref = male)	1.708*	1.794**	0.428	-0.351	1.581**
	(0.42)	(0.38)	(0.40)	(0.39)	(0.40)
**Education** (Ref = illiterate)					
Primary	-0.849	-1.968***	0.236	-0.560	-0.510
	(0.67)	(0.65)	(0.63)	(0.63)	(0.64)
Secondary	1.299**	1.455**	-1.259**	-0.261	-1.657***
	(0.64)	(0.63)	(0.61)	(0.61)	(0.62)
Technical	-0.520	-1.039*	0.033	-0.427	-0.592
	(0.56)	(0.54)	(0.53)	(0.56)	(0.53)
University or higher	-0.314	-1.607***	0.603	-0.298	-0.828
	(0.55)	(0.53)	(0.52)	(0.54)	(0.52)
**Number of years** (Ref= <5 years)					
5-10 years	-0.516	-0.708**	0.990***	-0.534*	0.025
	(0.316)	(0.311)	(0.318)	(0.310)	(0.311)
10-15 years	0.449	-0.405	0.838**	-0.211	-0.225
	(0.398)	(0.408)	(0.402)	(0.411)	(0.404)
>15 years	-0.320	-0.454	1.314***	-0.474	0.026
	(0.501)	(0.507)	(0.504)	(0.537)	(0.467)
**Ownership** (Ref= individually owned)					
Rented	-0.762**	0.546*	0.205	0.680**	1.126***
	(0.31)	(0.31)	(0.31)	(0.30)	(0.31)
Shared	1.965***	0.462	0.901*	-0.477	0.749
	(0.54)	(0.56)	(0.55)	(0.50)	(0.56)
**Production system** (Ref = household-bases)	0.851***	-0.758**	0.868***	0.082	-0.271
	(0.324)	(0.318)	(0.305)	(0.304)	(0.310)
**Number of years** (Ref= <5 years)					
5-10 years	-0.516	-0.708**	0.990***	-0.534*	0.025
	(0.316)	(0.311)	(0.318)	(0.310)	(0.311)
10-15 years	0.449	-0.405	0.838**	-0.211	-0.225
	(0.398)	(0.408)	(0.402)	(0.411)	(0.404)
>15 years	-0.320	-0.454	1.314***	-0.474	0.026
	(0.501)	(0.507)	(0.504)	(0.537)	(0.467)
**No. of permanent workers** (Ref = 1-5 workers)					
6-10 workers	0.220	0.127**	0.665**	-0.992**	0.681**
	(0.474)	(0.502)	(0.520)	(0.495)	(0.487)
>10 workers	1.664*	0.532	1.452	-1.431*	-0.670
	(0.854)	(0.920)	(0.883)	(0.835)	(0.901)
**No. of temporarily workers** (Ref = no temporary workers)					
1-5 workers	-0.080	0.344	0.569	0.503	-0.004
	(0.547)	(0.587)	(0.545)	(0.560)	(0.523)
6-10 workers	-0.022	0.730**	-0.88***	0.416	0.376
	(0.333)	(0.319)	(0.312)	(0.312)	(0.315)
**Total assets** (Ref = < 100 000 EGP)					
100000 - 200000	-0.993**	0.487	-0.152	-0.317	0.041
	(0.486)	(0.468)	(0.508)	(0.498)	(0.470)
200000 - 500000	-1.999***	0.412	-1.759**	-0.329	0.163
	(0.644)	(0.651)	(0.686)	(0.671)	(0.653)
500000-1000000	-2.786***	1.461	-0.161	0.572	-1.092**
	(0.993)	(0.966)	(0.957)	(0.948)	(0.894)
**Annual sales** (Ref = < 100 000 EGP)					
100000 - 300000	-0.428	-0.457	-0.408	-0.835	0.685

	(0.532)	(0.544)	(0.499)	(0.548)	(0.578)
300000 - 500000	0.009	0.635	-0.564	-0.515	0.541
	(0.673)	(0.736)	(0.678)	(0.690)	(0.686)
500000- 1000000	0.937	0.388	0.457	-0.405	0.180
	(1.126)	(1.030)	(1.018)	(1.035)	(0.973)
**Markets** (Ref = local)	0.941	0.149	0.283	-0.162**	0.013*
	(0.427)	(0.458)	(0.435)	(0.437)	(0.441)
**Coop. membership** (Ref = non-member)	0.229**	-0.577	0.286	0.555**	0.295
	(0.437)	(0.449)	(0.450)	(0.441)	(0.417)

Note: Standard errors are reported in parentheses. Dependent variables are *input availability* (**Model 1**), *production and operational costs* (**Model 2**), *Labor and human resources* (**Model 3**), Consumer demand & firm sales (**Model 4**), and *farm finances* (**Model 5**). Statistically significant at: *10, * *5, ***1 percent levels

Questionnaire

"Determinants of SARS-CoV-2 impact on small-scale commercial broiler production systems in Egypt: Implications for mitigation strategies"

Dear farm representative,

We are conducting a survey to investigate the effects of Coronavirus on small-scale commercial broiler farms in Egypt, where Al-Qalyubia Governorate is the case study. This survey is part of a larger scale project that we are implementing to assess the effects of the pandemic on the Egyptian food system and develop effective strategies for mitigating these effects and enhance preparedness and resilience of the Egyptian food system to future pandemics and natural hazard risks. The research team of this project consists of researchers from Egyptian and Swedish universities.

Your participation and your answers are very important to provide a good understanding of the situation of broiler production systems under the dynamics of the pandemic.

Please be noted that the information gathered in this survey will be treated confidentially and will be used for scientific research purposes **ONLY**. The responses provided will **NOT** be linked to individual names or addresses. All information that might indirectly identify respondents or farms will be eliminated from the data file before it is made available. Publications based on the data will never contain information that can identify individual respondents.

Your participation is strictly voluntary and you may refuse to participate at any time. If you have questions later, please contact me at XXXXX . If you would like to speak to our survey supervisor, you can call them at XXXXXXX.

Yours faithfully,

Dr. XXXXXXXXXXX

The project coordinator

CONSENT

Do you understand the purpose and nature of this study and agree to voluntarily participate in it by answering the questions in the survey?

If "**Yes**": I appreciate your willingness to help with this project.

I am an adult and I consent to taking this survey……………………………………..

If, "**No**": "Is this a bad time? Could you please let me know what would be a good time to talk?"

OR Still I do not consent taking this survey (skip to end)

**For Research team use only**	
**Form Number**	
**Date of the Interview**	
**Interviewer's Name**	

**Farm type** (***to be answered by the enumerator***):	Farm based ( )	Household based ( )

1. Respondent's and Farm's Characteristics

1.1 What is your position on the farm?
Owner ( ) Farm manager ( ) Agricultural engineer ( ) Veterinarian ( ) Other ( ), please mention: ……………..

1.2 Gender Female ( ) Male ( )

1.3 Into which age range do you fall? (Please tick one only)
18-24 years ( ) 25-34 years ( ) 35-44 years ( ) 45-54 years ( ) over 55 years ( )

1.4 Level of education
Illiterate ( ) Primary school ( ) Secondary school ( ) Technical qualification ( ) University degree or above ( )

1.5 How many years have you have you been working in the broiler farming business?...……………………

1.6 Farm ownership structure: Individually-owned farm ( ) Rented farm Shared farm ( )

1.7 Other than this farm, do you have any other farms? Yes ( ) No ( ), If yes, how many in total?…….

1.8 What is the number of permanent workers?

1.9 What is the number of seasonal workers?

1.10 What is the number of production cycles of your farm per year?

1.11 What are the main broiler strains in your farm? ……….

1.12 Where do you sell your farm production?

Local market ( ) Provincial market ( ) Local and provincial markets ( )

1.13 To which of the following categories the total assets of the farm belong?
<200 000 ( ) 200 000 - 500 000 ( ) 500 000 - 1 000 000 ( ) ≥ 1 000 000 ( )

1.14 To which of the following categories the total sales of the farm in 2019 belong?
<300 000 ( ) 300 000 - 500 000 ( ) 500 000 - 1000 000 ( ) ≥ 1 000 000 ( )

1.15 Are you a member of any farmer organization (e.g. producer associations, farmer networks) ?

Yes ( ) No ( )

2. Overall impact of Coronavirus on the surveyed farms

2.1. Overall, how did the containment measures that the government implemented to control the spread of Coronavirus affected your farm activities?

Very negative impact ( ) Negative impact ( ) No impact ( ) Positive impact ( ) Very positive impact ( )

2.2. At this point and compared to last year 2019, how would you assess the impact of the pandemic on the performance of your farm in relation to the following business dimensions?

Source of risk	Increase		Unchanged	Decrease	
	Increased	%		Decreased	%
Total turnover					
Total production cost					
Net profits					

2.3. Has your farm laid off any of your workers since the outbreak of the pandemic? No ( )Yes ( )

2.4. If yes, how many workers were laid off?............

2.5. Did the farm have to incur additional costs to cope with the pandemic? Yes ( ) No ( )

2.6. If yes, how would you estimate the cost of adaptation to the risks and challenges posed by the pandemic, as a percentage in your total earnings in 2020? Less than 5% ( ) 5-10% ( ) 10-20% ( ) 20-30% ( ) 30-40% ( ) 40-50% ( ) 50-60% ( ) > 60% ( )

2.7. Had you temporarily closed your farm due to the pandemic? Yes ( ) No ( )

2.8. If yes, for how many weeks /months was the farm closed?..................

2.9. Had you temporarily downsized the scale of your farm production due to the pandemic? Yes ( ) No ( )

2.10. If yes, for how many weeks /months did you downsize farm production?..................

2.11. What is the anticipated duration for your farm business to return to normal?
Less than 1 month ( ) 1-3 months ( ) 3-6 months ( ) 6-9 months ( ) 9-12 months ( ) more than a year ( ) Un able to judge ( )

2.12. How would you assess the capability of your farm to adapt to the impacts of the pandemic on farm production? Very capable ( ) Capable ( ) Somewhat capable ( ) incapable ( ) Very incapable ( )

3. Effects of Coronavirus on farm activities

Thinking of your farm performance in 2019 and considering the current situation of COVID-19, how does each of the following sources of COVID-19 risks represent a problem for your farm business?

**Sources of COVID-19 impact**	***How does this source represent a problem for your farm business?***					
	Not at all a problem	Minor problem	Moderate	Serious problem	Very serious	Unable to judge
Covid-19 has reduced total sales of the farm compared to 2019						
Due to the pandemic, the farm had to lay off workers and caused a loss of skilled labour						
Due to the pandemic, the farm is unable to pay financial obligations to traders and business partners						
Due to the pandemic, the cost of hiring workers (wages) increased						
The pandemic has caused a decline in the collection of external debts						
The pandemic caused inability of the farm to pay back farm loans						
The pandemic adversely affected worker productivity						
The pandemic reduced availability of vaccines and veterinary medicines						
Due to the pandemic, cost of feed increased						
The pandemic led to a loss of revenue from purchasing delays and reduced collection of receivables						

**Sources of COVID-19 impact**	***How does this source represent a problem for your farm business?***					
	Not at all a problem	Minor problem	Moderate	Serious problem	Very serious	Unable to judge
The pandemic increased financial stress on the farm and complicated access to finance						
The pandemic caused short supply of chicks on the market						
The pandemic has caused a difficulty in access to markets						
Mobility restrictions due to the pandemic, increased transportation cost						
The pandemic increased the risk of volatility in market prices						
The pandemic increased mortality rates of the cycles produced during the pandemic						
The pandemic increased cost of purchasing chicks						
The pandemic had decreased market demand						
The pandemic increased market rejections as products were refused by retailer						
Due to the pandemic, banks increased interest rate on new loans						
Due to the pandemic, cost of other tools and equipment needed for production increased						
The pandemic decreased the availability of feed						
The pandemic caused shortage of adequate feed of good quality						
The pandemic made it difficult for the farm to deliver contracted sales to partners due to lockdown						
Reduced availability of equipment used for collecting litter						
The pandemic increased the cost of vaccines and veterinary medicines						
The pandemic caused challenges for the farm w.r.t. the payment of farm rent and hire seasonal labor						
The pandemic increased rates of absenteeism						
Other, please specify:						
Other, please specify:						
Other, please specify:						

4. Risk management strategies

4.1. The following table includes a list of potential actions/ strategies that may be relevant to poultry farms to cope with the effects of the epidemic. To what extent do you agree with the fact that these strategies are appropriate for your farm in the current circumstances?

Strategy	Level of agreement				
	Totally agree	Slightly agree	Neutral	Slightly disagree	Totally disagree
We will seek loans from commercial banks					
We will seek government assistance programs					
We will apply for loans from small finance companies or from individuals					
We will negotiate with the lenders to avoid loan withdrawals					
We will add new partners or increase the farm capital					
We will reduce operating costs (for example, layoffs, salary cuts)					
We will take out insurance to cover unexpected events					
We will negotiate our contracts to settle them by mutual agreement					
We will seek a legal settlement for our unfinished contracts					
We will have to pay and settle the losses					
We will purchase inputs and production supplies from reliable suppliers that we have previously dealt with					
We will contact sellers and request an extension of the payment period					
We will close the farm or ward of the wards					
We will change operating strategies (production and marketing)					
We will purchase inputs from local markets					
We will contact other farms for coordination and assistance					
We will fulfil our orders by buying them from other companies and reselling them (subcontracts)					
We'll increase poultry prices to cover the higher costs					
We will introduce new poultry delivery options to our customers					
We will look for new suppliers and channels for purchasing production inputs					
We will focus on maintaining and improving the quality and weights of poultry					
We will sell in other (cities/governorates) places and open new markets					
We will reduce the number of employees					
We will reduce the salaries of the employees					
We will implement preventive measures and maintain a safe workplace					
We will increase wages to prevent employees from leaving the farm					

Other strategies, please specify:……………….					
Other strategies, please specify:……………….					

4.2. In your opinion, what are the most important elements that your farm possesses that may enable it to address the repercussions of the COVID-19 and its risks on farm operations?

Items	Totally disagree	Slightly disagree	Neutral	Slightly agree	Totally agree
Good governance and management systems					
Safety and quality of poultry					
Use of technological methods in production					
Skilled labor					
Capital and financial resources					
Farm proximity to marketing places					
Competitive price					
other					

5. Future follow-up

5.1. What is the full name of your company? (Optional)

5.2. We would greatly appreciate your participation in a follow up survey in a few months. If you'd like to participate, please leave your contact details (optional, mobile, email or landline number).

Thanks for your time and cooperation
